# Medical Imbroglio

**Published:** 1868-10-15

**Authors:** 


### *Medical Imbroglio.*

It is to be regretted that two of the faculty in one of the leading medical colleges of the West, have so far forgotten themselves as to have engaged in a pamphlet quarrel, in which pretty severe things have been said upon both sides. Having ourselves a profound and hearty distaste for professional polemics, we have carefully abstained from even the attempt to compass the merits of this particular case, for fear of becoming prejudiced in our opinions thereof and about. Individually, the contestants have earned the high respect and confidence of the profession throughout the country, and the Journal congratulates the faculty of the college with which they are connected, for their wise and prudent resolutions commanding the peace.

### *Bad Taste.*

We hear that the *Apostle*, probably smarting a little under the probable failure of the pet system of "Reform" which he has so long advocated, and *claims* to have inaugurated, had the questionable taste to allude to certain gentlemen, with whom he slightly disagrees, *by name*, in a recent discourse which he recently delivered to a small and select, yet nominally public, audience in a southern locality in this city. Aware, as we are, of the *Apostle's* somewhat inflammable temperament, and the attenuated nature of his cutaneous envelope — with the tenderest regard for his mental, moral and physical well-being, the JOURNAL begs of him, in his evangelico-medical *role*, not to attempt the Boanerges character, as it is not his best style — still worse to attempt the *Peter* line — swords are dangerous playthings. On the whole, would it not be better for our venerable friend to drop the apostle altogether, and try the older part — *Jeremiah?*

### *Note to the Editor.*

(*Extract from Editorial of Journal of August* 15*th.*)

"A correspondent wishes to know what kind of a pessary we prefer in our practice. We decline to answer — let each be persuaded in his own mind. In a tolerable experience of over twenty years, we have never recommended any kind, but what we have regretted it." The impression one would receive from this is, that all pessaries are useless.

Case 1st. Retroversion. The fundus lies low in the hollow of the sacrum and the os looks toward the symphysis pubis; after it is carefully replaced, it immediately falls back to its old position. Query—How would *you* manage the case?

Case 2nd. Fundus lying low in the hollow of sacrum, and the os looking nearly toward the coccyx. Retroversion with flexion. Restore it to its natural position, and the os looks towards the hollow of sacrum, but left to itself falls back immediately to its old position. Query—How will *you* treat it? If there is a better way than replacing the organ and keeping it in position by mechanical means, I desire to find it out, as I have a half dozen of just such cases on hand.

The Editor does *not* take the ground that in all cases "pessaries are useless." He does not take this ground, because he does not wish to be drawn into any controversy upon the subject.

He believes that pessaries sometimes relieve *effects*, never remove *causes*.

He believes they often do an immensity of mischief.

He believes that the weight of the wholly or partially enlarged uterus, upon which the displacement, etc., depends, may, in general, be relieved by medical and hygienic measures.

In his reply to the previous correpsondent, he merely gave his own experience.

He would treat the cases suggested by this correspondent according to what he found to be the matter.

